# A Blind Reversible Robust Watermarking Scheme for Relational Databases

**DOI:** 10.1155/2013/717165

**Published:** 2013-10-07

**Authors:** Chin-Chen Chang, Thai-Son Nguyen, Chia-Chen Lin

**Affiliations:** ^1^Department of Information Engineering and Computer Science, Feng Chia University, Taichung 40724, Taiwan; ^2^Department of Biomedical Imaging and Radiological Science, China Medical University, Taichung 40402, Taiwan; ^3^Department of Computer Science and Information Engineering, Asia University, Taichung 41354, Taiwan; ^4^Department of Information Technology, Travinh University, Travinh, Vietnam; ^5^Department of Computer Science and Information Management, Providence University, No. 200 Chung-chi Road, Taichung 43301, Taiwan

## Abstract

Protecting the ownership and controlling the copies of digital data have become very important issues in Internet-based applications. Reversible watermark technology allows the distortion-free recovery of relational databases after the embedded watermark data are detected or verified. In this paper, we propose a new, blind, reversible, robust watermarking scheme that can be used to provide proof of ownership for the owner of a relational database. In the proposed scheme, a reversible data-embedding algorithm, which is referred to as “histogram shifting of adjacent pixel difference” (APD), is used to obtain reversibility. The proposed scheme can detect successfully 100% of the embedded watermark data, even if as much as 80% of the watermarked relational database is altered. Our extensive analysis and experimental results show that the proposed scheme is robust against a variety of data attacks, for example, alteration attacks, deletion attacks, mix-match attacks, and sorting attacks.

## 1. Introduction

The rapid development of the Internet and related technologies has allowed for the tremendous ability to access and redistribute digital multi media contents. In such a context, protecting the ownership and controlling the copies of digital data have become very important. In the past few years, many research efforts have been dedicated to the development of approaches for protecting [1–4] and authenticating [5, 6] digital data. The watermarking technique [7–11] is one prospective solution to the aforementioned problems. Digital watermarking allows the user to embed an imperceptible watermark in the original data, that is, secret information such as a company logo that can be used to prove ownership of the data.

There are two basic types of watermarking techniques, that is, robust [12, 13] and fragile watermarking [14]. The first type of watermarking is robustness, which enables the watermarked data to resist a variety of malicious attacks and benign modifications of the user. The second type of watermarking is that a fragile watermark for tamper detection is used to identify and report every possible region in which someone has tampered with the watermarked data. This type of watermarking may be damaged or destroyed after processing is applied on the content of data either in incidental or malicious operations. Many watermarking techniques have been proposed for several types of digital data, that is, video, images, text, and audio [7–9, 15–19], and also for software and natural language text [10, 20]. A different way to categorize current watermarking schemes is to specify whether they are blind. Blind watermarking refers to the case in which the original image and the watermark are not required at the extraction phase [1, 5], whereas nonblind watermarking refers to the cases in which the original image and the watermark are required at the extraction phase [21].

Over the past 10 years, many scholars have focused their attention on watermarking techniques for relational databases [1–5, 11], because the use of database systems has been increasing a wide range of applications. However, most of the proposed watermarking schemes [1–3] have been irreversible, meaning that the original relational database cannot be recovered from the watermarked relational database. To solve this issue, scholars developed reversible watermarking techniques [5, 11] that allow the restoration of the spatial data in their original condition after the embedded watermark data have been extracted or detected. This capability is of critical importance for certain types of data, such as military and medical data, and it also can be applied to protect the ownership of relational databases. In addition, reversible watermarking techniques are also used to provide shareware or trial versions of database applications. In such cases, the original database can be reconstructed only when the user buys a license for a given application. 

The first well-known database watermarking scheme for relational databases was developed by Agrawal and Kiernan [1] for watermarking the numerical values in a relational database. Their fundamental assumption was that the watermarked relational database should be able to tolerate a small number of errors. This technique can be resistant to several attacks, such as alteration attacks, deletion attacks, mix-match attacks, and sorting attacks; also, it guarantees that the mean and variance of all numerical attributes will be minuscule. However, Agrawal and Kiernan's scheme cannot be used directly for embedding watermarks into categorical data because any bit change of a categorical value may make the value meaningless. To overcome this weakness in Agrawal and Kiernan's scheme, in 2004, Sion [4] proposed a watermarking technique that protects the rights to categorical data by modifying their current values to different values of the attribute if the change, which has an insignificant effect on the content, is acceptable in the categorical database. In 2008, Shehab et al. [2] presented a new watermarking technique that used the optimization-based technique. They divided the relational database into nonoverlapping partitions based on a secret key *K*. Then, the watermark bit was embedded into each partition by altering the partition statistics. Their scheme can be resilient to deletion attacks, alteration attacks, and insertions. Moreover, Shehab et al.'s scheme is efficient when the relational data in applications allow a small change in some of their values. In 2012, Farfoura et al. [5] designed a blind, reversible watermarking scheme by using a reversible data embedding technique called “prediction-error expansion” on integers to obtain reversibility. Their scheme completely detects the watermarked data with 100% accuracy, even when as much as 65% of the content of the watermarked relational database has been altered. Farfoura et al.'s scheme cannot completely withstand mix-match attacks, because it only can successfully detect the hidden watermark when less than 50% of the tuples that have been selected from other database sources are mixed with the current watermarked relational database. Moreover, Farfoura et al.'s scheme only embeds watermark bits into a fractional portion of the numerical attributes. If the numerical attributes do not contain the fractional portion, no watermark bits are hidden. In this paper, we propose a new, reversible, robust watermark scheme for relational databases to avoid the mentioned issues, to further improve the robustness of such databases against various forms of attacks, that is, alteration attacks, deletion attacks, mix-match attacks, and sorting attacks, and to prove their true ownership. To achieve reversibility, the APD reversible data hiding scheme, proposed by Li et al. [22], was used in the proposed scheme. When an owner suspects that someone has published a relational database that was copied illegally from her/his relational database *R*, he/she can use the watermark detection phase to detect or verify the embedded watermark data. 

The rest of this paper is organized as follows.  Section 2 presents a review of related work.  Section 3 gives a brief description of the watermarking model and the desirable properties of a watermarking system for a relational database. Then, the proposed watermarking scheme is presented in Section 4. In Section 5, we present our analysis of the robustness of the proposed scheme. The details of our experiments are presented in Section 6, and our conclusions and future work are given in Section 7.

## 2. Related Work

In the past decade, several reversible watermarking schemes designed for digital multimedia contents have been proposed based on difference expansion (DE) [16, 17] and histogram shifting [18, 19, 22]. Basically, schemes based on DE provide a larger embedding capacity, whereas the visual quality of the stego-image is better for schemes that are based on histogram shifting. In 2003, Tian introduced a reversible, DE-based watermarking scheme [16]. In Tian's scheme, the difference value between two neighboring pixels is calculated and doubled to embed one watermark bit. In 2007, Thodi and Rodríguez [17] proposed a watermarking scheme based on error prediction (PE) to hide the watermark data. In their scheme, a prediction technique is designed to predict the pixel value. Following that, the difference between the current pixel value and its predicted value is computed to embed the watermark data. These two schemes [16, 17] are based on the DE technique to achieve high embedding capacity. However, in such schemes, the pixels may have an overflow or underflow problem, and the visual quality of the stego-image is not very good. To increase the visual quality of stego-images, many researchers have proposed schemes based on the histogram-shifting approach. In 2006, Ni et al. introduced the first histogram-shifting scheme [18]. In their scheme, most of the pixels are shifted by one grayscale value to hide the watermark information. Their scheme achieves stego-images that have high visual quality, but the embedding capacity is limited. In 2009, Kim et al. [19] presented a reversible scheme based on a different histogram-shifting approach to obtain high capacity and imperceptible embedding by dividing the cover image into several subimages. The difference values between the sub-sampled images are calculated. Then, the difference values are shifted to embed more secret data. To further improve Kim et al.'s scheme, in 2010, Li et al. [22] proposed a reversible watermark scheme based on APD. To embed watermark data in Li et al.'s scheme, the difference sequence of pixels is computed as follows.

(1) $$\begin{array}{c} D_{i}=\{\begin{array}{cc} I_{i} & \text{if}\;i=0,\\ I_{i-1}-I_{i} & \text{if}\;1\leq i\leq n-1, \end{array} \end{array}$$

where *D*_*i*_ is the difference value, and *I*_*i*_ is pixel value. To embed watermark data, the histogram of difference sequence is generated, and the two pairs of peak points (PP) and the closest zero point (CZP), that is, (PP_1_, CZP_1_) and (PP_2_, CZP_2_), are selected from the generated histogram. If no closest zero point is determined in the histogram that has been generated, the APD scheme will select a minimum frequency point to serve the role of the zero point. Then, the minimum frequency point is cleaned to create the closest zero point. 

**Figure 1:**
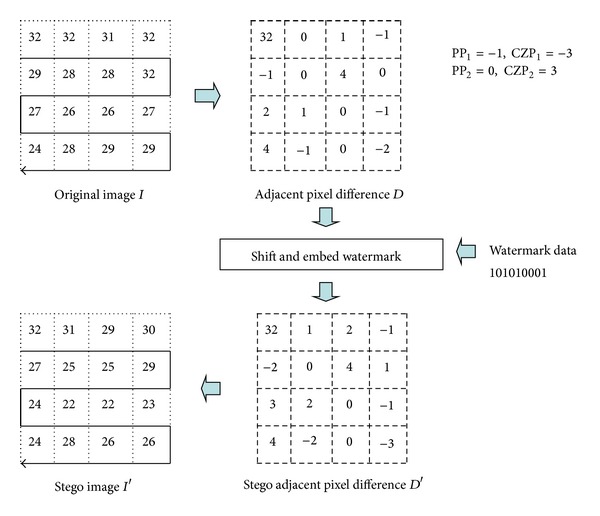
Example of watermark embedding of Li et al.'s APD scheme [22].

Since APD is used in our proposed watermarking scheme for relational database, we present an example in Figure 1 to explain the APD data-embedding concept. Let us assume that the original image *I* has its size as 4 × 4 pixels and that the watermark data are “101010001.” The adjacent pixel difference sequence *D* is calculated based on (1). Then, the two pixel difference pairs, that is, (PP_1_, CZP_1_) = (−1, − 3) and (PP_2_, CZP_2_) = (0, 3), are determined from the histogram of the difference sequence *D*. To embed the watermark data, the APD scheme shifts the difference values of *D* in range [CZP_1_, PP_1_) to the left-hand side of histogram by 1 and the values of *D* in range (PP_2_, CZP_2_] to the right-hand side of the histogram by 1. Since the current embedded watermark bit is *b*, if the value *d*_*i*_ of sequence *D* = −1 is the value of the first peak point PP_1_, the stego pixel difference value *d*_*i*_′ is calculated as *d*_*i*_′ = *d*_*i*_ − *b*. If the value *d*_*i*_ of sequence *D* = 0 is the value of the second peak point PP_2_, the stego pixel difference value *d*_*i*_′ is calculated as *d*_*i*_′ = *d*_*i*_ + *b*. Then, the stego image *I*′ is constructed from the stego pixel difference sequence *D*′ as shown in

(2) $$\begin{array}{c} I_{i}=\{\begin{array}{cc} D_{i}^{\prime } & \text{if}\;i=0,\\ I_{i-1}-D_{i}^{\prime } & \text{if}\;1\leq i\leq n-1. \end{array} \end{array}$$

In our scheme, we assume that the original image *I* is the last two digits of any numerical attributes to be selected for watermark embedding. In Section 4, we will present more details of the proposed watermarking scheme for a relational database.

## 3. Watermarking Model 

Assume that Alice is the owner of the relational database *R* that contains *n* tuples. To prove the ownership of relational database *R*, Alice should have the ability to extract the embedded watermark data *W*. The following properties should be satisfied [1, 5].

*(i) Blind System*. When the watermark data *W* are detected without the need of original relational database *R* and watermark data *W*. Only the embedding of the secret key *K* is required for both phases, that is, watermark embedding and watermark detection.

*(ii) Robustness*. The watermark should survive and be robust against a variety of kinds of data attacks, for example, benign database updating and malicious attacks.

*(iii) Imperceptibility*. The watermarked tuples and the watermarked attributes are selected randomly. Therefore, an attacker cannot determine which tuple and attribute were used to embed the watermark.

*(iv) Randomness*. Watermark data, *W*, are generated from the entirety of the information of the relational database to resist any localized attacks. 

*(v) Reversibility*. The original relational database *R* can be reconstructed completely after the watermark data *W* have been detected or verified. 

*(vi) Incremental Updatability*. Here, each tuple is selected for watermark embedding independently out of the rest of the tuples based on the cryptographic hash function.

*(vii) Prevent Illegal Embedding and Authentication*. The process is based on the secret parameters, that is, embedding secret key *K* and parameter *g*. Here, parameter *g* is the control parameter that determines the number of tuples selected for watermark embedding. Therefore, only the authorized owner who has all of these secret parameters can embed, verify, and detect watermark data. This can thwart attacker's attempts to verify the watermark data and to insert new watermarks into the watermarked relational database.

To avoid having the watermark data removed from the watermarked relational database when relational databases are updated or attacked, the following benign database updates and malicious attacks are discussed.

*(i) Benign Database Updates*. Following the scenario above, assume that one attacker has copied Alice's relational database *R* without knowing that this database has an embedded watermark data *W*. The attacker updates Alice's database when he/she uses it. Although the attacker had updated relational database *R*, Alice's watermark data would not be removed from *R*. 

*(ii) Alteration Attack*. An attacker tries to alter randomly some values of the tuples in the watermarked relational database with the aim of weakening the embedded watermark data.

*(iii) Deletion Attack*. An attacker tries to delete randomly some tuples in the watermarked relational database for the purpose of destroying the embedded watermark data. 

*(iv) Sorting Attack*. An attacker resorts the tuples of the watermarked relational data based on some attributes and hopes that the embedded watermark data cannot be detected. 

*(v) Mix-Match Attack (Insertion Attack)*. An attacker tries to mix the tuples of the watermarked relational database with tuples from other data sources hoping to delete the embedded watermark data.

## 4. The Proposed Watermarking Scheme

In this section, we will briefly introduce the concept of the proposed scheme in Section 4.1. Then, the proposed tuple selection, watermark embedding, and detection are presented in that order in the following subsections. 

### 4.1. Overview of the Proposed Scheme

**Figure 2:**
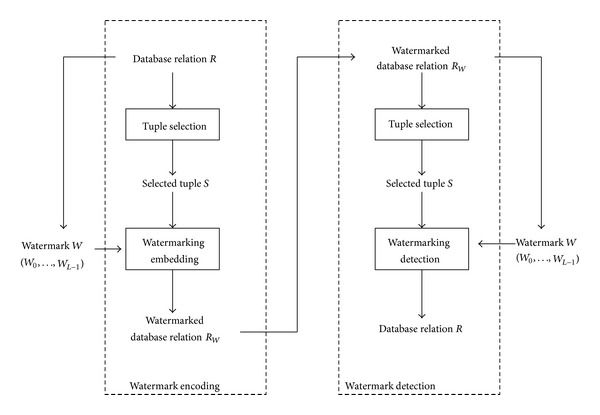
Flowchart of main processes in the proposed scheme.

After carefully considering Farfoura et al.'s scheme, we discovered that it embeds the watermark into the fractional portion of the numerical attributes in the relational database to minimize the distortion of the watermarked relational database. However, when the numerical attributes do not contain any of the fractional portion, no watermark bits are embedded. To overcome this problem, in this section, we introduce a new, blind, reversible, robust watermarking scheme. First, the watermark data are generated based on the information in the relational database. Then, the watermark data are embedded into the numerical attributes of the selected tuples instead of into the fractional portion of the numerical attributes.  Figure 2 shows the flowchart of the main processes in the proposed scheme.

Assume that the data set *R* is the relational database and that it is defined as *R* (*PK*, *A*_0_,…, *A*_*α*−1_), where *PK* is the primary key attribute, and any of the *A*_0_, …, *A*_*α*−1_ attributes are candidates for watermark embedding. To guarantee the security of the relational database *R*, we use the result of the one-way hash function, that is, computed by primary key *PK* and embedding secret key *K* to determine which tuples are selected for watermark embedding. The one-way hash function is defined as *h* = *H*(*M*), where *M* is an input message. The one-way hash function has three characteristics, that is, (1) given *M*, it is easy to compute *h*; (2) given *h*, it is difficult to compute *M* such that *H*(*M*) = *h*; (3) given *M*, it is difficult to find another input message *M*′ such that *H*(*M*) = *H*(*M*′). To meet the above requirements, many hash functions [23] can be considered for our scheme, that is, MD5 and SHA. A message authentication code (MAC) computed in (3) is a one-way hash function.  Table 1 shows the important parameters in our scheme. Consider

(3) $$\begin{array}{c} F\left(q\cdot PK\right)=H\left(K||q\cdot PK\right), \end{array}$$

where || indicates the concatenation function, and *q* · *PK* is the primary key attribute of the tuple *q* in relational database *R*. To increase security, the embedding secret key *K* could be chosen from a large key space, and it is only known by the owner. In addition, the watermark data *W* should be calculated by using the secure and random manner to counter various data attacks, such as alteration attacks, deletion attacks, mix-match attacks, and sorting attacks. To increase the security of the proposed scheme, the watermark data *W* will be generated by using (4) to counter guessing attacks for the embedded watermark data *W*:

(4) W=H(K||H   ×(K||DBname||Version||OID||DB inf⁡||…)),

where DB_name_ is the name of the database; Version is the version of the database; OID is the database owner's identity; DB inf⁡ is the database information, that is, the number of attributes, that is, the number of tuples of relational database *R*; *K* is the secret embedding key; and *H*() is a cryptographic hash function.

Figure 2 presents two phases of our proposed scheme, that is, watermark encoding and watermark detection. Watermark encoding can be summarized in the following steps.

*Step 1* (Tuple selection). Using the embedded secret key *K*, the tuples of relational database *R* are selected from the relational database *R* for watermark embedding. 

*Step 2* (Watermark embedding). Watermark *W* is embedded into selected tuples based on histogram shifting to generate the watermarked relational database *R*_*W*_′.

Assume that the owner suspects that one published relational database was copied illegally from her/his relational database. Then, the watermark detection algorithm is used to extract the embedded watermark from the suspected relational watermark *R*_*W*_′ and verify it. The watermark detection phase can be divided into the following two steps.

*Step 1* (Tuple selection). Using the embedded secret key *K*, the tuples are selected from the watermarked database relation *R*_*W*_′ for watermark extracting as that performed during the watermark encoding phase.

*Step 2* (Majority voting and watermark verification). The embedded watermark is extracted from the watermarked relational database for verification. In the watermark embedding phase, the watermark data *W* are embedded several times into the selected tuples of the relational database *R*. Thus, after the watermark data are extracted completely, several copies of each watermark bit can be obtained. Then, the majority voting mechanism is applied to determine the final watermark bit. Once the watermark data *W*′ have been reconstructed successfully, they are used for verification. 

In the following sections, each step is discussed in detail.

### 4.2. Tuple Selection

In this section, we present the tuple-selecting algorithm, called “Algorithm 1,” that was used to select the candidate tuples based on the secret embedding key *K*. For each tuple *q* ∈ *R*, the MAC value is computed using (3), and the value is used to determine whether a tuple is selected for watermark embedding. Based on the property of the one-way hash function, attackers cannot predict the selected tuples without knowing the secret embedding key *K* and parameter *g*. 

### 4.3. Watermark Embedding

In this section, we present the details of the watermark embedding process for selected tuple *S* of the relational database. The two basic functions used in our proposed scheme are as follows:

1. Get2digits(): used to extract the last two digits of numerical attribute *A*_*j*_ from selected tuples *S*,
2. GetMid(): used to sort number sequence and return the middle value of the sorted number sequence.

Algorithm 2 describes the process of embedding the watermark into the selected tuples *S* of relational database *R*. The Tuple_Selection() function, defined in Algorithm 1, is used to select the tuples from relational database *R* for watermark embedding. Using the Get2digits() function, the last two digits are extracted from the numeric attribute *A*_*j*_ of the selected tuples *S* to generate a number sequence *Seq*. This means that the number sequence *Seq* contains |*S*| integers. Then, the GetMid() function is used to sort the number sequence *Seq* and determine the middle value *Mid* from the sorted sequence. When *Mid* is obtained, the Dif() function is used to subtract each value in the number sequence *Seq* from *Mid* and to return the difference sequence *Dif_Seq*. Then, the histogram of difference sequence, *Dif_Seq*, is generated, and two pairs, that is, (PP_1_, CZP_1_) and (PP_2_, CZP_2_), of this histogram are generated. Here, we extended Li et al.'s scheme [22], where the peak point, PP_1_ or PP_2_, is determined in the difference sequence *Dif_Seq*, and one watermark bit is embedded. After completing the process of embedding the watermark, the Reflect_Update_Att() function is used to update the new value for each of the selected attributes in the relational database. The watermark embedding algorithm is shown in Algorithm 2. 

In Algorithm 2, to enhance the security of the proposed scheme, the candidate attribute *A*_*j*_ of each selected tuple is determined as shown in line 3 of Algorithm 2. The corresponding MAC value for each attribute *A*_*j*_, computed as shown in (3), is used to determine whether an attribute can be selected for watermark embedding. To embed more watermark bits, more attributes can be selected for each tuple. To guarantee the reversibility of the proposed watermarking scheme, some parameters, that is, *K*, *g*, *Mid*, and two pairs, that is, (PP_1_, CZP_1_) and (PP_2_, CZP_2_), are recoded for watermark detection.

### 4.4. Watermark Detection

Assume that Alice suspects that Bob has illegally copied or tampered with her watermarked relational database *R*_*W*_. We assume that Bob did not drop the primary key attribute or modify the values of the primary keys because they contain valuable information. Therefore, modifying this information will reduce the usefulness of the relational database.

In this section, the detection algorithm for relational database *R*_*W*_′ is discussed. To extract and verify the embedded watermark data, we must know the parameters used for watermark embedding, including *K*, *g*, *Mid*, and two pairs, that is, (PP_1_, CZP_1_) and (PP_2_, CZP_2_). The watermark detection algorithm starts by selecting tuples from the database relation *R*_*W*_′ based on the embedding secret key *K* and the primary attribute *PK*. The selected tuples *S* are reconstructed, and the candidate attributes *A*_*j*_ of each tuple are also determined in the same manner as in the watermark embedding phase. When obtaining the selected tuples *S*, we can extract the embedded watermark and recover the original relational database *R*. Since the watermark data *W* are embedded into the relational database *R* several times, several copies of each watermark bit can be obtained after the watermark detection algorithm is processed completely. Then, the majority voting mechanism technique is used to determine the final watermark bit. Here, we will count the numbers of its values to be ones or zeroes, respectively. If the number of ones is larger than detection parameter *τ*, then the final watermark bit is one; otherwise the final watermark bit is zero.  Algorithm 3 shows the details of watermark detection for watermark relational database *R*_*W*_′.

## 5. Robustness Analysis

In this section, we analyze the robustness of the proposed watermarking scheme against malicious attacks and benign database updates, which were mentioned in Section 3. There, we used an analysis that was similar to that of Farfoura et al.'s scheme [5], in which the authors extended Agrawal and Kiernan's scheme [1] to propose a blind, reversible scheme for watermark relation data. 

Assume that the attacker does not know any of the secret information used in embedding the watermark, including the embedding secret key *K* and parameters, that is, *g*, *α*, *Mid*, and two pairs, that is, (PP_1_, CZP_1_) and (PP_2_, CZP_2_). Therefore, the attacker does not know which tuples were selected for embedding the watermark. 

A robust watermark scheme must survive all malicious data attacks or benign database update operations that may destroy or adversely affect the watermark embedded in the relational database. Such attacks can be classified into four types, that is, alteration, deletion, mix-match, and sorting attacks. The related analysis of our proposed scheme for these four types of attacks is demonstrated in the following subsections. 

### 5.1. Alteration Attack

In the alteration attack, attacker Bob tries to remove the embedded watermark by altering randomly the data value of *β* tuples of the watermarked relational database. We assume that Bob does not know any secret information. Therefore, he cannot know which tuples were selected for embedding the watermark. 

**Figure 3:**
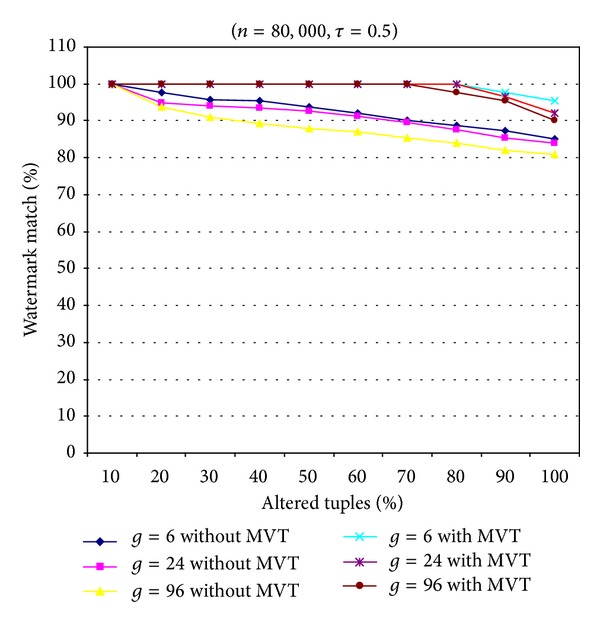
Resilience to alteration attacks with and without MVT for different values of parameter *g*.

Figure 3 shows the performance of our scheme against an alteration attack. Two types of proposed schemes, that is, the proposed scheme with and without the majority voting mechanism technique (MVT), were tested with different values of parameter *g*. Figure 3 shows that the proposed scheme with MVT obtained better resilience to the alteration attack than that without MVT. In addition, the smaller the value of parameter *g* was, the higher the number of tuples selected for watermark embedding became. Figure 3 shows that the proposed scheme with MVT (for *g* = 6) can detect the watermark data successfully when more than 80% of the tuples were altered randomly. With the majority MVT, the watermark detection of our scheme only fails to reconstruct the watermark bit *w*_*i*_ when it was smaller than *n*_*wi*_/2 times of the embedded watermark bit *w*_*i*_ extracted from the watermarked relational database matched, where *n*_*wi*_ is the number of times the watermark bit *w*_*i*_ was embedded into the selected tuples.

**Figure 4:**
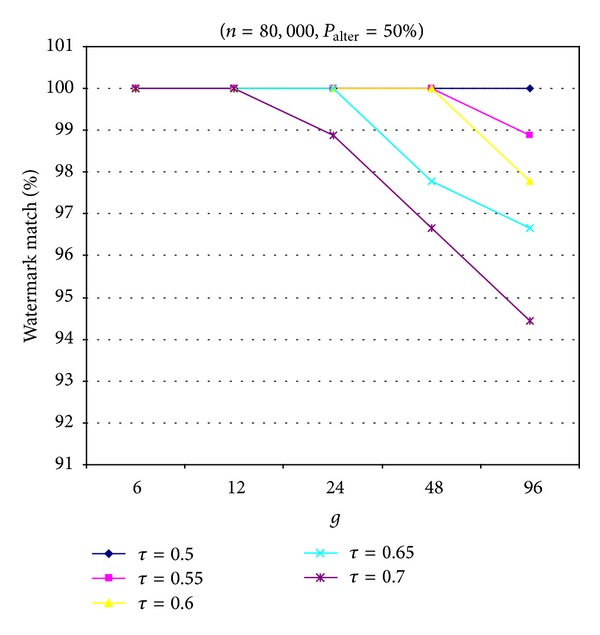
Relation between parameter *g* and detection parameter *τ* of our proposed scheme with MVT in an alteration attack.

Figure 4 presents the relation of the parameter *g* and the detection parameter *τ* of our proposed scheme with MVT for an alteration attack. When the relational database *R*_*W*_ contained 80,000 tuples, the probability *P*_alter_ that the rate of tuples was altered successfully in this attack was 50%, and the detection parameter *τ* was in the range of 0.5 to 0.7. We can observe easily that, with smaller values of parameter *g* and detection parameter *τ*, we can obtain a higher ratio of watermark match. In other words, the proposed scheme with MVT achieved greater robustness against alteration attacks when smaller values of parameter *g* and detection parameter *τ* were used.

### 5.2. Deletion Attack

**Figure 5:**
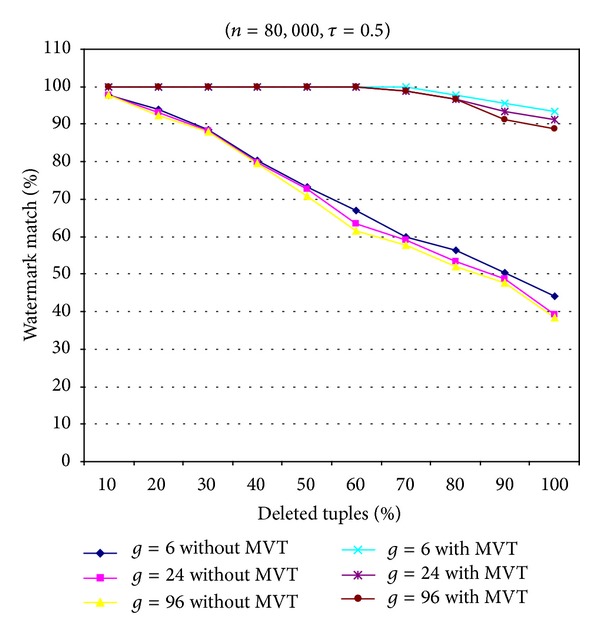
Resilience to the deletion attack.

In this attack, attacker Bob randomly drops *β* tuples from the watermark relational database *R*_*W*_. We assume that the attacker does not know any secret information, thus he/she cannot know which tuples were selected for embedding watermarks.  Figure 5 clearly shows that, at the smaller values of parameter *g*, the proposed scheme achieves greater resilience to the deletion attack. Furthermore, our scheme obtained better results for this attack when MVT was used. In Figure 5, it is easy to see that the embedded watermark data were successfully extracted with 100% accuracy, even when up to 70% of tuples were deleted in the proposed scheme with MVT (for *g* = 6). This is because the proposed scheme used the MVT technique, and the watermark data were embedded into the relational database several times. When the watermark detection was processed completely, the MTV technique was used to determine the best watermark data. Conversely, the proposed scheme without MVT (for *g* = 6) only extracted successfully and correctly 98% of the watermark data, when 10% of the tuples of watermark relational database *R*_*W*_ were deleted.

### 5.3. Mix-Match Attack

**Figure 6:**
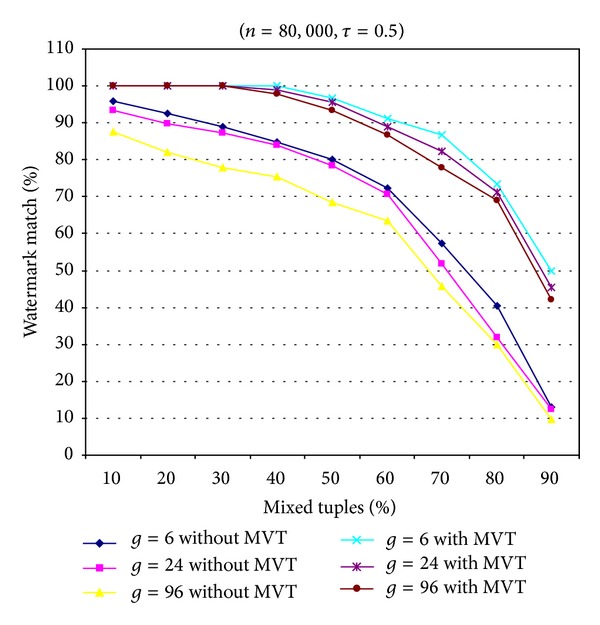
Resilience to the mix-match attack.

In this attack, also referred to as an insertion attack, attacker Bob tries to weaken the embedded watermark data by mixing the watermark relational database *R*_*W*_ with the number of tuples from other data sources to generate a new relational database with the same size as *R*_*W*_.  Figure 6 shows the results of our proposed scheme against the mix-match attack, which were obtained by randomly selecting difference ratios of the other data source and mixing them with those of the watermark relational database *R*_*W*_. It is easy to see that, when the mixing rate was 50% and the detection parameter *τ* = 0.5, the watermark match rate of the proposed scheme with MVT (for *g* = 6) was 97%. However, the watermark match rate of the proposed scheme without MVT (for *g* = 6) was only 80%. Note that, when the proposed scheme (for *g* = 6) was used with the MVT technique, the attacker would have to use more than 80% of tuples from other sources with those of the watermarked relational database *R*_*W*_ in order to destroy 30% of the embedded watermark data. However, when MVT was not used in the proposed scheme (for *g* = 6), the attacker only had to use 60% of tuples from other sources to mix with *R*_*W*_ to remove 30% of the embedded watermark data. 

### 5.4. Sorting Attack

In our proposed schemes with and without MVT, each tuple was processed independently. The pseudo hash value of the primary key of each tuple and the embedding secret key *K* were used to determine the selected tuples for watermark embedding and the index of the corresponding watermark bit for both phases, that is, watermark embedding and watermark detection. Therefore, our proposed scheme can withstand sorting attacks.

## 6. Experimental Results

To show the performance of our proposed scheme, our experimental results are presented in this section, and our results are compared to the results of two existing schemes, that is, Shehab et al.'s scheme [2], and Farfoura et al.'s scheme [5]. All experiments were performed on a PC with an Intel(R) Core i7-3770 CPU @ 3.4 GHz and a 8 GB RAM. The operating system used for testing was Windows 7 Professional 64 bit. In this paper, all algorithms were programmed by Microsoft Visual Studio 2005 C# using the ADO component to visit the Microsoft SQL Server database. We used our algorithm to generate artificial relational database *R* of nine attributes, one of which contained the primary key attribute, and the other eight contained the numerical attributes. The eight numerical attributes were considered as candidates for being embedded as watermark data in the three schemes. The size *n* of the generated relational database *R* was 80,000 tuples. The size of the watermark data *L* = 60, the parameter *g* = 6, and the detection parameter *τ* = 0.50 were used in our experiments. To ensure the accuracy of the results of each experiment we conducted, each test was repeated 100 times. Then, for each trial, the average was computed of all of the successful watermark matches. 

**Figure 7:**
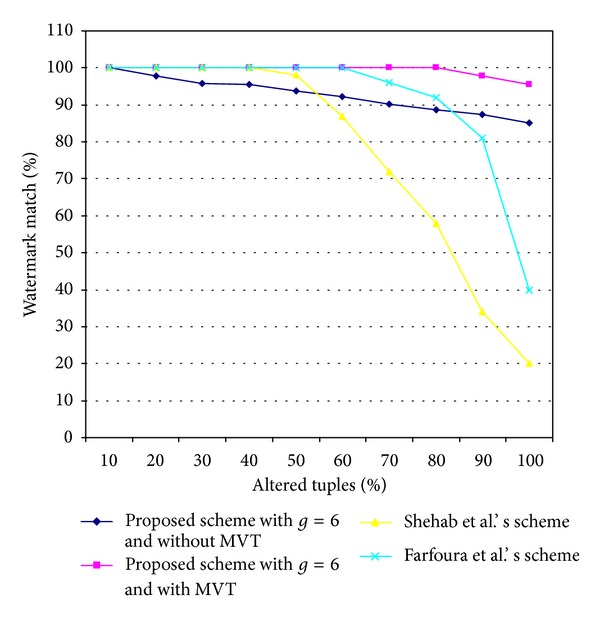
Comparison of the results for resilience to an alteration attack by the two proposed schemes and two other schemes.

Figure 7 presents the results of the resilience to an alteration attack of the proposed scheme with and without MVT, Shehab et al.'s scheme [2], and Farfoura et al.'s scheme [5]. Figure 7 shows that, among the four schemes, the proposed scheme without MVT was the worst. This was because the MVT was not used in this scheme. However, the proposed scheme with MVT was stronger in resilience to an alteration attack than the other three schemes. Even when up to 80% of the tuples of the watermarked relational database *R*_*W*_′ were altered, the proposed scheme with MVT recovered the watermark data with 100% accuracy. Shehab et al.'s scheme [2] and Farfoura et al.'s scheme [5] were able to reconstruct the watermark data with 100% accuracy only when the tuples of *R*_*W*_′ were altered by 40% and 60% or less, respectively. 

**Figure 8:**
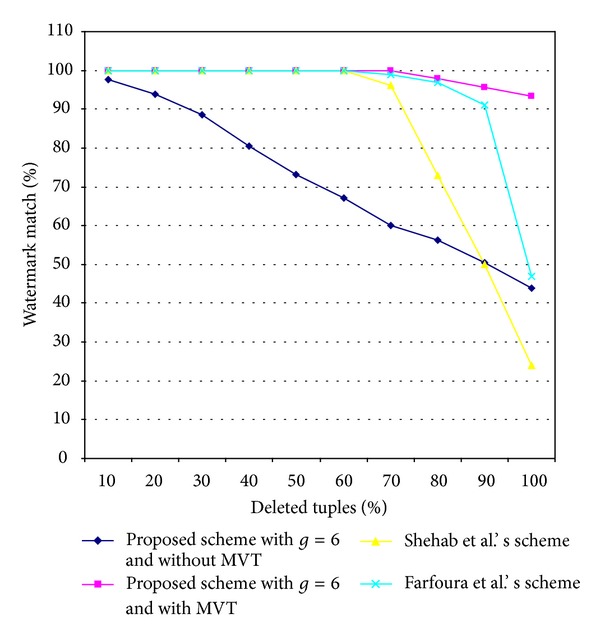
Comparison of the results for the resilience to the detection attack of the two proposed schemes and two other schemes.

Figure 8 shows the results of resilience to a deletion attack for the four schemes. Obviously, the proposed scheme with MVT was more robust in its resilience to a deletion attack than the other three schemes. When using our scheme with MVT, the watermark data were extracted with 100% correctness, even when more than 70% of the watermarked relational database *R*_*W*_′ was deleted. The proposed scheme with MVT obtained the strongest resilience to the deletion attack and the alteration attack because it randomly selected each tuple and attribute based on the one-way hash function of the embedding secret key and the corresponding primary key of tuple for watermark embedding. When sufficient key space of the embedding secret key *K* was used, it was more difficult for attackers to alter or delete the embedded watermark bit from the watermarked relational database. In addition, in the proposed scheme with MVT, these attacks were weakened further by the repetition of the embedding watermark data and by the use of the MVT. 

**Figure 9:**
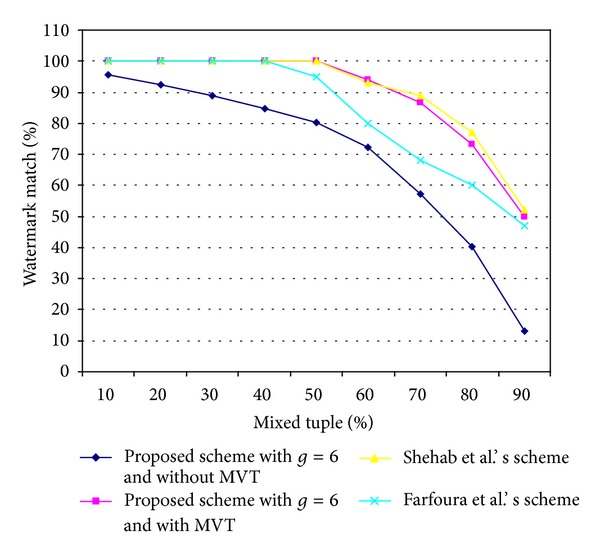
Comparison of results for resilience to a mix-match attack of the two proposed schemes and the other two schemes.

Figure 9 shows results for the resilience to a mix-match attack for the four schemes. Figure 9 shows that Shehab et al.'s scheme and our proposed scheme with MVT obtained the stronger resilience to a mix-match attack. These schemes were able to extract the embedded watermark data with 100% of accuracy when up to 50% of tuples from other sources were mixed with the watermarked relational database. Shehab et al.'s scheme had the better robustness to this attack because the relational database in Shehab et al.'s scheme was divided into partitions. All tuples in each partition were processed to embed one watermark bit instead of a single tuple. Therefore, the effect of inserting tuples is only a minor perturbation in Shehab et al.'s scheme. Our scheme with MVT also obtained high robustness to the mix-match attack because it randomly selected the tuples and attributes for watermark embedding. In addition, in the proposed scheme based on the MVT technique, the watermark data were embedded repeatedly into the relational database, and the MVT was used for reconstructing the watermark data *W*′.

## 7. Conclusions

In this paper, we presented a new, blind, reversible, robust watermarking scheme for a relational database. The proposed schemes were designed to protect the ownership of the database. In addition, the true owner achieves the full reconstruction of the original relational database after the watermark data have been detected and extracted. The experiments showed that the proposed scheme with MVT was resilient to various attacks. Moreover, comparisons between our proposed scheme and two existing schemes indicated that the performance of the proposed scheme with MVT was superior to those of the other two schemes. Based on the experimental results and our analysis of the robustness of the schemes, we concluded that the proposed scheme with MVT was more secure and robust than the two existing schemes we tested. 

In the future, we aim to extend the proposed scheme for being used as a fragile watermarking technique. Further studies should be conducted in the nonnumeric domain, that is, categorical and alphabetic attributes.

## Figures and Tables

**Algorithm 1:**
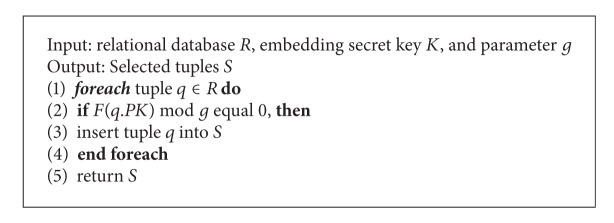
Tuple selection.

**Algorithm 2:**
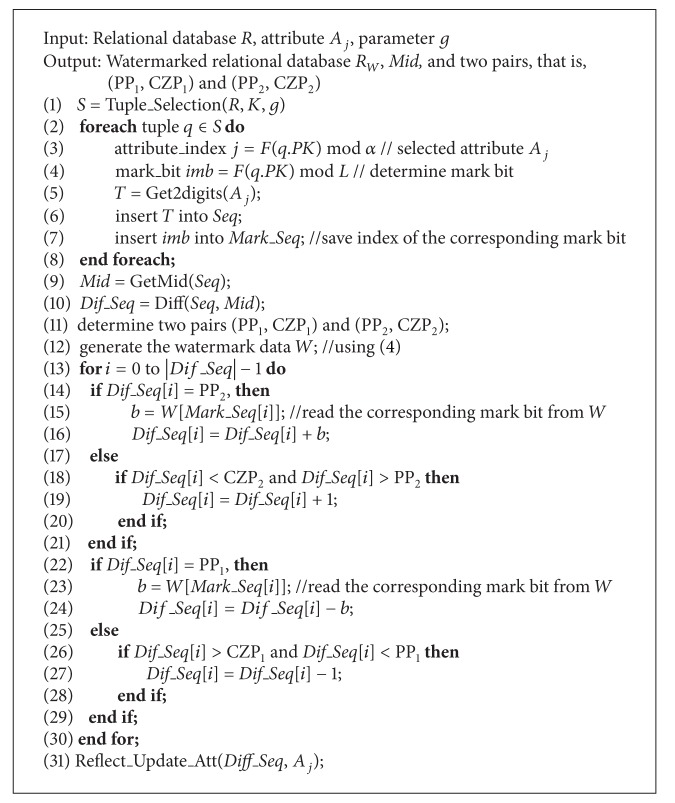
Watermark embedding.

**Algorithm 3:**
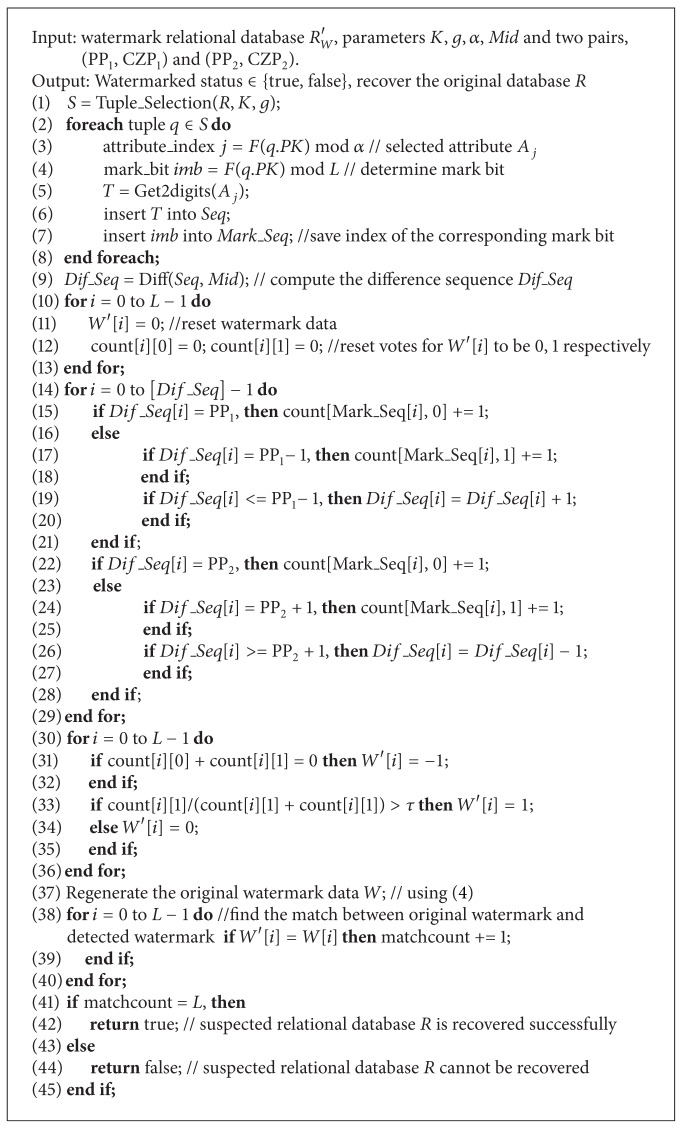
Watermark detection.

**Table 1:** Notations used in our scheme.

Parameters	Descriptions
*R*	Database relation to be watermarked
*R*_*W*_	Watermarked relational database
*A*_*j*_	Attribute *j* in relational database *R*
*n*	Number of tuples in relational database *R*
PP_*i*_	The *i*th peak point of the histogram
CZP_*i*_	The *i*th closest zero point of the histogram
*K*	The embedded secret key
1/*g*	Fraction of tuples selected for watermark embedding
*α*	Number of attributes in the relational database available for embedding watermarking
*L*	The length of watermark data *W*
*τ*	Detection parameter
